# Coaching-Based Teleoccupational Guidance for Home-Based Stroke Survivors and Their Family Caregivers: Study Protocol for a Superior Randomized Controlled Trial

**DOI:** 10.1155/2022/9123498

**Published:** 2022-08-22

**Authors:** Li Zhang, Yanning Yan, Zengxin Sun, Xinjing Ge, Xiaolu Qin, Keh-Chung Lin

**Affiliations:** ^1^Graduate School, Hebei Medical University, Shijiazhuang 050051, China; ^2^Department of Rehabilitation Medicine, Hebei General Hospital, Shijiazhuang 050051, China; ^3^School of Occupational Therapy, College of Medicine, National Taiwan University, Taipei 10617, Taiwan

## Abstract

**Background:**

Home-based rehabilitation has been shown to be useful for stroke survivors to participate in daily life activities and return to their families. However, many home-based stroke survivors face challenges in the lack of professional guidance, rational training plans, and insufficient motivation, which will affect their rehabilitation outcomes to varying degrees. Though occupational therapy and coaching are widely recommended for stroke rehabilitation, studies that combine these two interventions via telerehabilitation in home-based rehabilitation are limited. Hence, this study will explore whether coaching-based teleoccupational guidance (CTG) will help stroke survivors and caregivers obtain satisfactory outcomes.

**Methods:**

This single-blind (assessor), two-arm parallel superior randomised controlled trial will be conducted in the Hebei General Hospital, Shijiazhuang, China. Ninety-two participant dyads in home-based rehabilitation will be recruited and randomised to either CTG (intervention group) or a standard telerehabilitation group (control group). Participant dyads in the intervention group will follow a 6-step circle procedure and receive 12 teleoccupational coaching sessions over 3 months via WeChat. Data will be collected at baseline, after the intervention (3 months), and follow-up (6 months). The Reintegration to Normal Living Index will be the primary outcome to assess the participation of stroke survivors. Secondary outcomes will not only involve an observation of changes in activities of daily living, intrinsic motivation, motor function, and quality of life of stroke survivors but also will focus on the caregivers' perceived benefit and care burden. *Discussion*. This trial will assess the effects of CTG compared with standard telerehabilitation. We believe that the results of this study will add to the understanding of occupational therapy for stroke survivors in home-based rehabilitation and provide a reference for developing health policy and facilitating other chronic management. *Trial Registration Number*. The Chinese Clinical Trial Registry ChiCTR2200061107.

## 1. Introduction

Stroke has become the leading cause of mortality and morbidity in adults of China [1, 2]. Approximately 70%–80% of stroke survivors experience varying degrees of impairment of their motor, sensory, cognitive, and other functions [3] that might reduce their participation, decrease their quality of life, and increase the caregivers' burden [4]. Home-based rehabilitation, an effective complement to in-hospital rehabilitation, is essential for realizing the stroke survivors' full potential to live better [5]. As one of the core pillars of home-based rehabilitation, occupational therapy is an important intervention to help stroke patients return to their families and society. However, owing to the lack of professional guidance, reasonable training plans, insufficient motivation, poor compliance, and so on, the quality of usual home-based rehabilitation is unsatisfactory [4, 6, 7]. Therefore, the active exploration of efficient home-based rehabilitation strategies has great practical significance.

Coaching is a multidimensional, cognitive behavioural intervention that promotes client-centred, goal-oriented development through various engagements, such as interviews, listening, questioning, and the inculcation of other skills to enhance self-monitoring behaviours and a sense of responsibility, cultivation of internal motivation, skill acquisition, and to improve the health status and quality of life [8, 9]. In 2007, the Canadian occupational therapists association recommended coaching as a practical skill in the occupational therapy practice framework. Although considerable evidence supports the effectiveness of coaching on the self-management quality of life in chronic disease [10, 11], few studies have investigated coaching combined with occupational therapy in home-based stroke survivors. In addition, some previous related studies only added simple coaching strategies to intervention processes, which might not reflect the true effect of the coaching [12, 13].

The aim of this study is to investigate the effectiveness of coaching-based teleoccupational guidance (CTG) for home-based stroke survivors and their caregivers. It is anticipated that, compared to routine telerehabilitation, CTG would lead to better rehabilitation outcomes for stroke survivors and their caregivers.

## 2. Materials and Methods

### 2.1. Study Design

**Figure 1:**
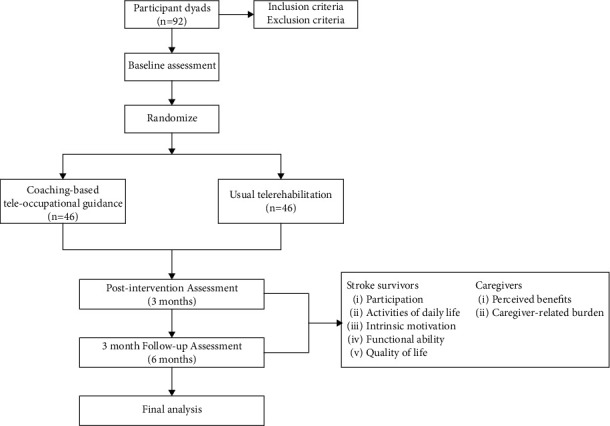
Study flow chart.

This single-blind (assessor), two-arm parallel superior randomised controlled trial of a CTG program in comparison with standard telerehabilitation for stroke survivors and their caregivers will be conducted at the Hebei General Hospital in Shijiazhuang, China (Figure 1). The Hebei General Hospital is one of the earliest large polyclinics that set up a Rehabilitation Medicine Department on the Chinese mainland and is equipped with sufficient, high-quality rehabilitation resources. There will be sufficient patient supply in this research setting. The duration of the intervention and follow-up in this study will be 3 and 6 months, respectively. The Ethics Committee of Hebei General Hospital approved the study protocol, which follows the Standard Protocol Items: Recommendations for Interventional Trials (CONSORT Additional File [1]. Informed consent from all participants will be obtained prior to their enrolment in the study.

### 2.2. Participants

#### 2.2.1. Inclusion and Exclusion Criteria for Stroke Survivors and Caregivers

In this trial, we will recruit stroke survivors and their primary caregivers as participant dyads subject to their provision of written informed consent. Participants will be included based on the following inclusion criteria: (1) men or women aged 18 to 70 years; (2) diagnosed with ischaemic or haemorrhagic stroke either by computerized tomography scanning or magnetic resonance imaging; (3) being more than 6 months after the onset of stroke; (4) modified Rankin scale (MRS) score of 2 to 4 points; (5) undergoing home rehabilitation and no admission plan within 3 months; and (6) residing within or around the city of Shijiazhuang. Patients will be excluded if they (1) have a Glasgow coma scale (GCS) score of less than 15; (2) have cognitive and (or) psychotic disorders; (3) have severe comorbidities, including circulatory, digestive, immune, and haematological disorders; (4) have other diseases of the locomotor system, such as fracture, severe osteoporosis, and osteoarthritis, that will influence the stroke survivors' motor function; (5) have bilateral brain lesions; and (6) have severe sensory dysfunction.

Caregivers will be included if they meet the following inclusion criteria: (1) men or women who are at least 18 years old; (2) are the primary caregivers and can ensure the time and support of participating in the patient's home rehabilitation; and (3) can operate WeChat.

#### 2.2.2. Inclusion Criteria for Occupational Therapists

The selection of qualified occupational therapists is crucial for ensuring trial quality. We will select occupational therapists who have the following qualifiication: (1) an educational background in occupational therapy, (2) engaged in occupational therapy for at least 5 years, and (3) strong interpersonal skills.

### 2.3. Recruitment Strategy

Using the medical record checklist of the Rehabilitation Medicine Department, a research assistant will screen the stroke survivors who meet the prespecified inclusion criteria and do not meet the exclusion criteria. If stroke survivors and their primary caregivers meet the study's eligibility requirements, the researcher will contact them via telephone to share relevant information about the study. Participant dyads who are interested to participate in the study will be invited by the principal researcher (YNY) for a detailed face-to-face interview to assess their eligibility to participate in the study.

### 2.4. Randomisation and Blinding

Participant dyads will be allocated to the CTG or the standard telerehabilitation group by using 1 : 1 randomisation sequences that are generated by IBM SPSS Statistics version 25. The random number sequence generation will be undertaken by a research assistant who is not involved in the assessment and intervention and will be placed in sequentially numbered, opaque, sealed envelopes for blinded group allocation.

Stroke survivors, primary caregivers, and occupational therapists will not be blinded due to the nature of the intervention. However, the assessor will be blinded to collect each outcome measure and undertake data entry. Furthermore, all participant dyads will meet with the same trained and blinded study assessor at all-time points to ensure consistency of outcome measures.

### 2.5. Description of the Interventions

#### 2.5.1. Coaching-Based Teleoccupational Guidance

**Figure 2:**
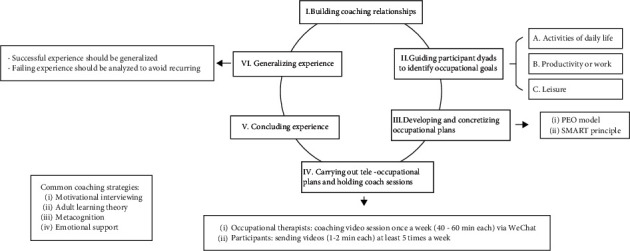
The general cycle procedure of the CTG intervention.

We will conduct the intervention according to a 6-step cycle (Figure 2).

*(1)* Step I*: Building Coaching Relationships.* The treating therapists will establish equal, friendly, and mutual trust coaching relationships with the participant dyads. A private WeChat group that includes only the occupational therapist, the patient, and the caregiver will be provided to ensure timely guidance and communication in home-based rehabilitation. Occupational therapists will train participants to use WeChat's functions, such as dialling videophones, sending videos, and the methods of clicking photos or capturing videos. If WeChat has technical difficulties due to network problems or other factors, the telephone call will be used as a backup communication method.

*(2)* Step II*: Guiding Participant Dyads to Identify Occupational Goals.* The treating therapist will use the Canadian occupational performance measure (COPM) and coaching techniques to guide participant dyads to identify up to 5 occupational goals that reflect a wide range of ADLs (e.g., eating a meal with chopsticks and spoon; dressing, and grooming); productivity (e.g., resuming paid employment); community engagement (e.g., going to the weekly market; resuming driving), domestic activities (e.g., cooking a family meal); and leisure activities (e.g., walking the dog; restoring furniture) [14]. Moreover, the therapist will use the motivational interview of coaching strategies flexibly (e.g., emotional support, expressing empathy, “looking forward,” “looking back,” and so on) to guide the patients to think and describe the reasons for making changes and to clarify the gap between their current situation and goals, which can help participants to gain intrinsic motivation for change.

*(3)* Step III*: Developing and Concretizing Occupational Plans.* The person–environment–occupation (PEO) model will be used to help occupational therapists work with participant dyads to identify a structured and individualised problem-solving plan. In conjunction with the use of the PEO model, plans should involve the following 4 aspects: (a) occupational therapists will provide therapeutic occupational activities, ADLs, and social skills training to improve personal functioning based on the stroke survivor's career goals and performance, cultural background, hobbies, and family environment; (b) according to their needs, participants will be provided professional suggestions for environmental adjustment to gain safety and a convenient home environment (e.g., installing toilets and handrails), whereas importantly focussing on support from caregivers (e.g., creating a positive family environment, training care skills); (c) we can adjust occupational activities if necessary (e.g., converting offline shopping to online shopping); (d) participant dyads will receive individualised content education where appropriate, including ergonomic, skills remediation, safe management, compensatory strategies, and alternative methods. To standardise the plan, we will comply with the principles of SMART (Specific, Measurable, Attainable, Relevant, and Time-bound).

*(4)* Step IV*: Carrying Out Teleoccupational Plans and Holding Coaching Sessions.* Treatment plans will be posted on WeChat as scheduled. Occupational therapists will provide participants with a weekly coaching video session (40–60 min each) via WeChat for 3 months. We will follow the “ORAG” (Open-ended questions, Reflective listening, Affirmation, and Guide) program (Table 1) to structure the sessions.

In addition, participants will be asked to send videos (at least 5 times a week) to the WeChat group. The content of these videos should include the practice of occupational exercises, participating in ADLs, and so on. If the researchers have any questions or detect potentially unsafe behaviours in the videos, they can give suggestions or adjust timely occupational activities by sending voice messages and pictures or conducting video calls on WeChat. Furthermore, if participants have doubts about the study, they can contact the researchers at any time through the WeChat group.

*(5)* Step V*: Concluding Experience.* An occupational therapist will evaluate the achievement of goals and encourage participant dyads to summarise their experience to help them build up the ability to independently deal with problems.

*(6)* Step VI*: Generalising Experience and Entering the Next Cycle.* Finally, the successful experience should be affirmed and generalised, whereas the failure experience should be analysed to avoid recurrent failures. Participants enter the next cycle to achieve other goals when a goal has been completed. Though the CTG process is depicted as a cyclic progression (Figure 2), each step may be revisited at any time.

### 2.6. Standard Telerehabilitation (Control Group)

Participants in the standard telerehabilitation group will receive home-based rehabilitation plans according to the selected occupational goals of the stroke survivors and have a weekly video session (40–60 min each) with the occupational therapist via WeChat for 3 months, which aims to help participant dyads to adjust plans and solve problems. Participants should send videos (1-2 min each) of home-based rehabilitation to the WeChat group at least 5 times a week for 3 months. Researchers will ensure timely follow-up and adjust the participants' training via WeChat.

### 2.7. Outcomes Measures

**Figure 3:**
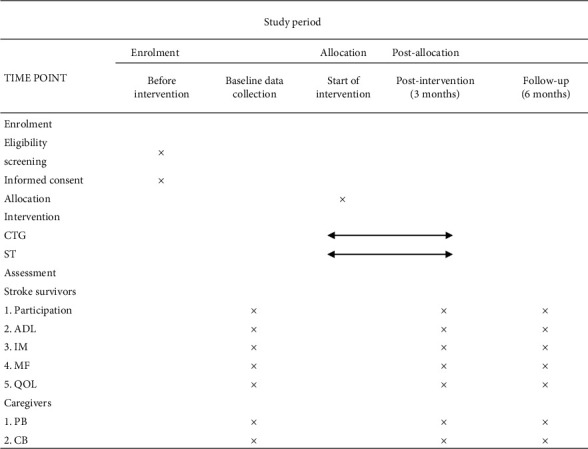
The figure shows the enrolment, interventions, and assessment time points. CTG, coaching-based teleoccupational guidance; ST, standard telerehabilitation; ADL, activities of daily living; IM, intrinsic motivation; MF, motor function; QOL, quality of life; PB, perceived benefits; CB, caregiver-related burden.

The participant dyads will visit our outpatient department for outcome evaluation at baseline, immediately after the intervention, and at the 6-month follow-up. Sex, age, type of stroke, hemisphere, dominant side, profession, and other social information will be collected after enrolling participants in this trial. Based on the purpose of the trial, we choose the following parameters as the study's outcomes. Moreover, most of the items are simple questions-and-answers and included patients who are in relatively good health condition, stroke patients, and their caregivers take only approximately 30 minutes to complete the assessment. But if patients' cannot complete all measures at once, the researcher who is in charge of the assessment can use WeChat videos to finish the remaining part the next day.  Figure 3 shows the study's timeline.

### 2.8. Outcomes of Stroke Survivors

#### 2.8.1. Primary Outcomes

Participation: The Reintegration to Normal Living Index (RNLI) is an 11-item self-report scale to test participation in 2 dimensions: physical activities and social events. Scores on the self-report scale range from 1 to 110, with higher scores indicating better participation. The RNLI has been translated into Chinese and has been proved to be reliable and valid for the Chinese population (Cronbach's *α* 0.92; retest reliability coefficient 0.87) [15].

#### 2.8.2. Secondary Outcomes

ADL: We will measure this outcome using the Modified Barthel Index (MBI) and Lawton Instructive Activities of Daily Life (Lawton IADL). The MBI consists of a 10-item scale to test basic ADLs. Scores range from 0 to 100, with higher scores indicating better independence [16]. The Lawton IADL has 8 items of activities that test instructive ADLs, with each scored from 0 to 2 [17]. The MBI and Lawton IADL have been widely used in stroke rehabilitation studies.

Intrinsic motivation: We will measure this outcome using the Intrinsic Motivation Inventory (IMI). The IMI consists of 54 items of self-report questionnaires to test motivational structures for targeted activities in 6 dimensions (score range: 0–7): interest/enjoyment, perceived competence, pressure/tension, and value/usefulness [18].

Motor function: We will measure this outcome using the Fugl–Meyer Assessment-Upper Extremity (FMA-UE), Motor Activity Log, and 6-minute walking test (6MWT). (1) The FMA-UE is a 33-item scale that is used to assess the movement, coordination, and reflex actions of the shoulder, elbow, forearm, wrist, and hand. The total score ranges from 0 to 66 points, with higher scores indicating better motor function [19]. (2) The Motor Activity Log will be used to test the extent of natural use of the affected upper limb in the real world (outside the rehabilitation setting) in persons who have suffered a stroke. The scale has 2 subscales: quality of movement (QOM) and amount of use (AOU). Overall, it consists of 30 items for assessing the quality and quantity of the use of the affected limb for daily tasks [20]. (3) The 6MWT is a simple test to evaluate the walking function; it is a familiar form of exercise for patients and is more relevant to their everyday lives, reflecting participation and walking capacity [21].

Quality of life: We will measure this outcome using the stroke-specific quality of life scale (SS-QOL). The SS-QOL is a 49-item self-report scale to test the quality of life in 12 dimensions: energy, family roles, language, mobility, mood, personality, self-care, social roles, thinking, upper extremity function, vision, work/productivity, each scored from 1–5, with a higher score indicating well-being [22].

### 2.9. Outcomes of Caregivers

Perceived benefits: We will measure this outcome using the Caregiver Benefit Finding Scale (CBFS). The CBFS was designed by Chinese scholars with a Cronbach's *α* range of 0.885 to 0.953 for the subscales. It contains 26 items in 4 dimensions: individual growth, health promotion, family growth, and self-sublimation. The total score ranges from 26 to 130 points, with better scores indicating more perceived benefits [23].

Caregiver-related burden: We will measure this outcome using the Chinese version of the Zarit Caregiver Burden Interview (ZBI-c). The ZBI-c contains 22 items in 2 dimensions: personal strain and role strain. The total score ranges from 0 to 88 points, with better scores indicating a more significant caregiver burden, and the scale was validated in the Chinese population with a Cronbach's alpha of 0.87 [24].

### 2.10. Sample Size

The RNLI, commonly used as a reliable outcome measure in occupational therapy research, will be employed for sample-size estimation. According to our preliminary test and previous studies, the minimum significant difference between the 2 groups in the T2–T1 interval for the RNLI in the survey is 14 points [25]. The standard deviation of the measure was reported as 21 points in previous studies [25]. Setting the *α* at 0.05 (two-tailed) and the power at 80% and assuming a drop-out rate of 20%, the minimum sample size for each arm is 46. Based on this estimation, we will recruit at least 92 participant dyads (92 participants and 92 caregivers in each group–intervention versus standard telerehabilitation) for the study.

### 2.11. Statistical Analysis

An intention-to-treat (ITT) analysis will be used for data analysis, as specified by CONSORT guidelines [26]. Statistical analysis will be performed using IBM SPSS Statistics version 25. Descriptive analysis will be used to analyse the demographic data in each participant group. The Shapiro–Wilk test will be used to assess the normality, and the data will be presented as mean ± standard deviation. In contrast, skewed data will be reported in the median and interquartile range. The categorical data will be expressed as a proportion (percentages). Comparisons of demographic and clinical characteristics at the baseline of both the study groups will be tested by independent sample *t*-tests and the chi-square or nonparametric Mann–Whitney *U*-test, respectively.

Repeated-measures analysis of variance (ANOVA) will be used to analyse the primary and second outcomes with regard to time (baseline, 3-month, and 6-month follow-up) as the within-group factor and interventions (standard telerehabilitation versus coaching-based teleoccupational guidance) as the between-group factor. We will evaluate the main effects of time, group, and time-group interaction effects. The Mauchly test will be performed to check the sphericity of the data; if significant, the Greenhouse–Geisser statistic will be used to adjust the degrees of freedom [27]. The level of significance will be set at 0.05.

### 2.12. Data Management

The medical data recording sheets, signed informed consent forms, and the data of the WeChat group (photos, videos) from participant dyads will be stored securely. The principal researcher (YNY) will be responsible for monitoring data in this study. Only authorised research assistants will have access to the trial data. In addition, as this study is an early, exploratory, short-term study without severe safety concerns, the Independent Data Monitoring Committee will not be set up.

### 2.13. Quality Control

Before commencing the study, the principal researcher (YNY), a senior occupational therapist who is adept at using coaching in home-based rehabilitation, will provide 3 workshops (1 hour each) for 2 occupational therapists (ZXS, XJG) and 2 occupational therapist assistants (XLQ and LZ) who will be involved in the intervention to introduce and train them on the related information about this study. The first workshop will inform occupational therapists of the trial flow, division of research, and associated issues of quality control. The second workshop will enable them to master the commonly used coaching strategies and home-based occupational therapy for stroke survivors. The team will recruit 4–6 inpatient stroke patients for the final workshop, which undertakes the role of pre-experiment to help occupational therapists practice the acquired knowledge and get feedback from patients, peers, and the principal researcher. Through the pre-experiment, researchers can discover the problems existing in the trial and solve them in time. In addition, occupational therapists will be divided into 2 groups, each group consisting of 1 occupational therapist who is the primary implementer of the study and 1 occupational therapist assistant who cooperates with the occupational therapist to complete daily work and follow the process of study. Specifically, XLQ and ZXS as a group will be in charge of 23 participant dyads in CTG and 23 pairs in the control group (46 participants and 46 caregivers in total) using a random number sequence, as will the other group of LZ and XJG. And the principal researcher (YNY) will pay close attention to their work and provide help if necessary. To ensure the accuracy of the trial data, two postgraduates who will not participate in this study will enter data independently.

### 2.14. Intervention Fidelity

The occupational therapist will document the content of the coaching home-based rehabilitation by taking notes and session video recordings to monitor intervention fidelity. The principal researcher will test fidelity by rating 3 randomly selected video recordings of sessions per participant.

### 2.15. Dealing with Contingencies

The first possible contingency in the study is the difficulty in recruiting an adequate number of stroke survivors to meet the sample-size requirement. If necessary, expanding the scope of recruitment to the departments of neurology, neurosurgery, or other hospitals in Shijiazhuang should be considered. The second possible unexpected problem is the high drop-out rate of participants. To prevent this possibility, researchers will improve patients' correct perception of clinical trials and establish appropriate expectations. In addition, if the participants feel uncomfortable during the intervention period, researchers will pause the intervention or provide them with appropriate treatment.

## 3. Discussion

Coaching, which emerged from motivational interviewing, is the practice of health education and health promotion within a coaching context to enhance the well-being of individuals and facilitate the achievement of their health-related goals [28, 29]. Motivational interviewing, a core component of coaching strategies, focuses on motivating the patient to achieve goals that enhance the quality of life and improve the outcome of rehabilitation [28]. In our study, motivational interviewing methods will be adopted for stroke survivors and caregivers, such as emotional support, expressing empathy, supporting self-efficacy, looking forward, looking back, and so on. These strategies will equip them with knowledge and motivation to change behaviours or to identify advantages and barriers for participant dyads to achieve goals in the current situation [30, 31]. Furthermore, coaching sessions are crucial to ensure positive health outcomes, which can help the occupational therapist find and solve timely problems that happen to patients and their caregivers. Coaching sessions will be carried out by structured procedures, including open-ended questions, reflective listening, affirmation, guiding, and summary, which are easier to conduct for occupational therapists who, by their vocation, are nonprofessional coaches [32]. Most importantly, this process will enable the participant dyads to learn to independently solve relevant problems in future home-based rehabilitation.

Many fundamental coaching principles are congruent with occupational therapy values and commitment to client-centred practice [33]. In our study, the occupational therapist will use the COPM model, which is widely used in stroke rehabilitation research, to help participants identify goals. Goal setting should be individualised and realistic to motivate participants to achieve their goals. However, some previous home-based rehabilitation programs paid more attention to physical functions but ignored the demands, and psychological and social functions of stroke survivors, which could not bring a long-term effect [34]. In contrast, researchers focus on the demands of patients and make activities of daily living integrate into occupational plans so that they can quickly return to home life and maintain a long-term effect. According to recent reports, approximately 80% of stroke survivors only participate in a few meaningful activities, which decreases their quality of life and increases the caregivers' burden to different degrees [35]. Participation is the ability to engage in meaningful activities, which is considered a significant outcome measure, especially for home-based stroke survivors [36]. Therefore, enhancing the participation of stroke survivors is prioritised by occupational therapists. Furthermore, researchers choose the RNLI as the primary outcome in this study because it has advantages in assessing overall participation of stroke from the perspective of daily functioning and perception of self and considering the person's satisfaction with the present situation, which is the best fit with the purposes of our study. However, there is relatively little research about participation in stroke. It is anticipated that the results of this intervention will provide related evidence to inform peers in home-based rehabilitation.

In addition, caregivers play an important role in home-based rehabilitation, which are not only in charge of taking care of stroke patients and assisting and monitoring patients participating in rehabilitation training but also bear substantial mental pressure and psychological burden [37, 38]. In this study, we focus on the role and burden of caregivers and listen to their demands. Moreover, the occupational therapist will provide emotional support and stroke-related knowledge for caregivers by CTG in order to improve quality of life and decrease burdens. In terms of perceived benefits, CBFS is chosen to measure this outcome, which takes individual growth, health promotion, family growth, and self-sublimation into consideration and supports caregivers to realize their growth and find personal meaning during the caregiving experience.

Occupational plans for stroke survivors are crucial to enable them to gain well-being. However, making occupational plans is challenging for occupational therapists. Therefore, we combined the PEO model with the SMART principle to optimise both. First, the PEO model will guide occupational therapists to analyse residual function, support available, and degree of challenge and clear the key points of plans from different aspects of the person, the environment, and the demands of the occupation [39]. Second, it is a complex but essential requirement for researchers to set plans. Some of the characteristics of programmes that effectively alter behaviours should be considered. The SMART principle will satisfy this requirement and play a vital role in structuring and quantifying plans to ensure that participants achieve their goals [40].

The delivery of an intervention needs to be safe, private, and easy to use. WeChat is a free software created by the Tencent Technology Company to provide instant messaging services for clients, which supports the quick sending of free voice messages, videos, and pictures as well as voice and video calls. The app is highly popular in China and has the advantages of easy operability in real-time while being convenient, fast, and economical. In the home-based rehabilitation period, stroke survivors may experience various confusing problems. Therefore, timely communication is essential for both occupational therapists and participants. At least 5 videos each week, video call sessions once a week, and needs-based WeChat contact will allow occupational therapists and participants to solve problems or adjust plans without delay.

Several vital steps affect study fidelity. First, researchers have developed standardised and logical intervention procedures based on coaching theory, occupational therapy strategies, and behavioural change techniques. Second, training interveners and documenting the content of the intervention will improve adherence to the study design. Third, stroke survivors will be asked to send pictures and videos regularly to monitor the completion of the plan mutually. In addition, we will offer a questionnaire survey to participants to assess their general satisfaction across various aspects of the intervention and collect data on the number of participants who drop out of the study to analyse reasons for quitting.

The protocol has some limitations. First, because of the nature of the intervention, it is impossible to blind the occupational therapists and participants. Moreover, emotional support is a crucial aspect of CTG. However, occupational therapists have to provide this support due to professional ethics when stroke survivors in the control group ask for help. Therefore, both possibilities may increase the risk of bias. Second, successful home-based rehabilitation is a sustainable long-term process whereas our observation period is for 6 months. It is expected that we can explore the efficacy of a longer duration of the intervention and follow-up. Finally, future studies should take economic effects into account.

## Figures and Tables

**Table 1:** The “ORAG” program for CTG.

Items	Topics
Open-ended questions	Occupational therapists will guide participants to think about whether the occupational performance of this week was moving towards their goals by asking open-ended and enlightening questions.
Reflective listening	Occupational therapists should listen to the feedback information of the participants and analyse the relevant emotional changes and thoughts.
Affirmation	Occupational therapists should praise the progress made by the participants and encourage them to analyse and summarise their successful experiences.
Guide	If participants have barriers to achieving goals, occupational therapists will help them independently analyse possible and potential reasons. Next, suggestions and resources will be provided to identify bridges to success by combining the adult learning model and occupational strategies.

## Data Availability

Not applicable.

## Abbreviations

CTG: Coaching-based teleoccupational guidance
MRS: Modified Rankin scale
GCS: Glasgow coma scale
COPM: Canadian occupational performance measure
ADL: Activities of daily living
PEO: Person–environment–occupation
RNLI: Reintegration to normal living index
MBI: Modified Barthel index
Lawton IADL: Lawton's instructive activities of daily life
IMI: Intrinsic motivation inventory
FMA-UE: Fugl–Meyer assessment-upper extremity
6MWT: 6-Minute walking test
QOM: Quality of movement
AOU: Amount of use
SS-QOL: Stroke-specific quality of life scale
CBF: Caregiver benefit finding scale
ZBI-c: Chinese version of the Zarit caregiver burden interview
ITT: Intention-to-treat
ANOVA: Analysis of variance.

## Supplementary Materials

Supplementary Materials Additional file 1: CONSORT 2010 checklist of information to include when reporting a randomised trial.
